# Transcriptional Response of *Staphylococcus aureus* to Sunlight in Oxic and Anoxic Conditions

**DOI:** 10.3389/fmicb.2018.00249

**Published:** 2018-02-23

**Authors:** Jill S. McClary, Alexandria B. Boehm

**Affiliations:** Civil and Environmental Engineering, Stanford University, Stanford, CA, United States

**Keywords:** *Staphylococcus*, sunlight, photoinactivation, transcription, RNA, sequencing

## Abstract

The transcriptional response of *Staphylococcus aureus* strain Newman to sunlight exposure was investigated under both oxic and anoxic conditions using RNA sequencing to gain insight into potential mechanisms of inactivation. *S. aureus* is a pathogenic bacterium detected at recreational beaches which can cause gastrointestinal illness and skin infections, and is of increasing public health concern. To investigate the *S. aureus* photostress response in oligotrophic seawater, *S. aureus* cultures were suspended in seawater and exposed to full spectrum simulated sunlight. Experiments were performed under oxic or anoxic conditions to gain insight into the effects of oxygen-mediated and non-oxygen-mediated inactivation mechanisms. Transcript abundance was measured after 6 h of sunlight exposure using RNA sequencing and was compared to transcript abundance in paired dark control experiments. Culturable *S. aureus* decayed following biphasic inactivation kinetics with initial decay rate constants of 0.1 and 0.03 m^2^ kJ^−1^ in oxic and anoxic conditions, respectively. RNA sequencing revealed that 71 genes had different transcript abundance in the oxic sunlit experiments compared to dark controls, and 18 genes had different transcript abundance in the anoxic sunlit experiments compared to dark controls. The majority of genes showed reduced transcript abundance in the sunlit experiments under both conditions. Three genes (*ebpS, NWMN_0867*, and *NWMN_1608*) were found to have the same transcriptional response to sunlight between both oxic and anoxic conditions. In the oxic condition, transcripts associated with porphyrin metabolism, nitrate metabolism, and membrane transport functions were increased in abundance during sunlight exposure. Results suggest that *S. aureus* responds differently to oxygen-dependent and oxygen-independent photostress, and that endogenous photosensitizers play an important role during oxygen-dependent indirect photoinactivation.

## Introduction

In the United States, pollution of recreational waters led to 23,481 beach closures in 2011 (National Resources Defense Council, 2012), and contact with polluted recreational waters can cause gastrointestinal illness, respiratory infections, and skin ailments (Cabelli et al., 1982; Haile et al., 1999; Colford et al., 2007). To prevent excess exposure to microbial pollution, recreational waters are traditionally monitored by the detection of culturable fecal indicator bacteria (FIB), such as *Escherichia coli* and enterococci, which requires processing times of ~18–24 h (US EPA, 2012). However, FIB concentrations are known to fluctuate on short timescales due to factors such as sunlight exposure and tides (Boehm et al., 2009; Russell et al., 2013; Corsi et al., 2016), calling into question the utility of FIB measurements that require long processing times. To address this issue, rapid detection methods and water quality modeling techniques have begun to be applied in recreational water quality monitoring (Wade et al., 2008; Thoe et al., 2015; He et al., 2016; Tryland et al., 2016). However, an incomplete understanding of the mechanisms leading to bacterial decay in coastal environments limits our ability to include these factors in water quality models and points to a need for improved understanding of these mechanisms.

Photoinactivation, or inactivation due to sunlight exposure, is an important process that modulates bacterial concentrations in environmental waters (Sassoubre et al., 2015) and can occur by both direct and indirect mechanisms. Direct photoinactivation involves the absorption of photons by vital cell components, like nucleic acids, which leads to cellular damage (Sinha and Häder, 2002). In contrast, during indirect photoinactivation, photons are absorbed by sensitizers (either endogenous or exogenous to the cell) which become excited and subsequently damage vital cell components either directly or through generation of reactive oxygen species (ROS) (Curtis et al., 1992). Several studies have identified ROS as one of the most important factors influencing photoinactivation of both bacteria and viruses in natural waters (Kohn and Nelson, 2007; Sassoubre et al., 2012; Maraccini et al., 2016b). However, the relative importance of direct and indirect photoinactivation mechanisms in environmental systems remains poorly understood. In engineered systems, advanced oxidation unit processes, which combine UV treatment with ROS or ROS precursors, are being increasingly considered for use in wastewater reuse treatment trains (Sun et al., 2016). The combination of ROS precursors and light exposure is also the basis of photodynamic therapy, which can be used for localized treatment of bacterial infections (Sabbahi et al., 2008). Due to the importance of photoinactivation in a range of contexts, a better understanding of direct and indirect photoinactivation mechanisms is needed.

Investigation into the transcriptional response of bacteria to sunlight stress can provide insights into photoinactivation mechanisms. Microarrays and RNA sequencing (RNA-seq) have been used to investigate the effects of sunlight exposure on gene expression in FIB, including *Enterococcus faecalis* (Sassoubre et al., 2014) and *E. coli* (Berney et al., 2006; Al-Jassim et al., 2017). A range of cellular processes are triggered by sunlight exposure, including DNA repair, oxidative stress response, virulence, and SOS response (Berney et al., 2006; Sassoubre et al., 2014; Al-Jassim et al., 2017). Evidence to date suggests that different species transcribe different genes in response to sunlight exposure. For example, following sunlight exposure, genes coding for superoxide dismutase, a highly conserved enzyme involved in oxidative stress response, were identified as upregulated in *E. faecalis* (Sassoubre et al., 2012, 2014) but downregulated in *E. coli* (Berney et al., 2006; Al-Jassim et al., 2017). This information allows us to gain insight into cells' ability to repair or respond to sunlight exposure and advances our understanding of bacterial fate in sunlight-exposed waters.

One bacterial pathogen of concern in recreational waters is *Staphylococcus aureus*, which is commonly detected in recreational beach water and sand (Charoenca and Fujioka, 1993; Goodwin et al., 2012; Levin-Edens et al., 2012; Hower et al., 2013) and can cause gastrointestinal, respiratory, and skin infections. Epidemiological studies have identified associations between recreational water contact and various skin ailments (Wade et al., 2008; Yau et al., 2009; Sinigalliano et al., 2010). Some studies have further identified relationships between staphylococci concentrations in beach water and skin ailments (Prüss, 1998), and between *S. aureus* skin infections and recreational water contact (Charoenca and Fujioka, 1995), indicating that recreational beaches may be a reservoir for pathogenic *S. aureus* in the environment. Recently, concern regarding particular strains of antibiotic resistant *S. aureus* that are able to spread within the community has grown. Compared to healthcare-associated strains, community-associated *S. aureus* have also been shown to be more virulent in mouse models, partially due to their ability to resist ROS-mediated killing by neutrophils (Voyich et al., 2005).

The present study investigates the transcriptional response of *S. aureus* suspended in clear seawater to sunlight exposure in order to gain insight into photoinactivation mechanisms and bacterial stress response. Experiments were performed under both oxic and anoxic conditions in order to differentiate between photostress responses associated with oxygen-mediated and non-oxygen-medated photoinactivation mechanisms. To our knowledge, this is the first study to evaluate genome-wide transcriptional response of a pathogenic bacterium under both oxygen-dependent and oxygen-independent photostress conditions.

## Materials and methods

### Photoinactivation experiments

*Staphylococcus aureus* photoinactivation under oxic and anoxic conditions was evaluated using an experimental design identical to a previously published study (McClary et al., 2017). In brief, *S. aureus* subsp. *aureus* str. Newman (ATCC 25904) was grown in chemostat cultures filled with 20 mL 25% Brain Heart Infusion (BHI) broth (Fluka Analytical, Steinheim, Germany). *S. aureus* was grown in chemostats in order to improve reproducibility between experimental replicates (Maraccini et al., 2015). After reaching a stable growth rate, bacteria were washed twice and resuspended in ~1 L sterile simulated seawater for a concentration of ~10^7^ CFU/mL. The composition of simulated seawater was derived from Parker et al. (2013) and consisted of 424 mM sodium chloride, 0.87 mM sodium bromide, 29.2 mM sodium sulfate, 0.27 mM sodium carbonate, 1.83 mM sodium bicarbonate, 10.5 mM potassium chloride, 54.8 mM magnesium chloride, and 10.7 mM calcium chloride. The initial concentration of ~10^7^ CFU/mL of *S. aureus* was chosen to allow for sufficient masses of mRNA to be extracted for sequencing. For experiments performed under anoxic conditions, the bacteria-seawater suspension was divided into two black PVC pipe reactors (described previously McClary et al., 2017), one experimental and one control. Reactors were sealed by fixing quartz glass plates to the top of the reactors with silicone sealant and were then sparged with nitrogen through rubber septa to remove oxygen from the water column and headspace. After sparging for ~30 min, reactors were held in the dark at 15°C with constant stirring for 12 h to acclimate to a cool, oligotrophic environment. For experiments performed under oxic conditions, reactors were set up identically but with quartz glass plates secured loosely with tape and without nitrogen sparging.

After 12 h of incubation at 15°C, the experimental reactor (oxic or anoxic) was placed in a 15°C recirculating water bath in a solar simulator (Atlas Suntest XLS+; Chicago, IL) equipped with a 1.1 kW xenon arc lamp and a glass filter to generate full spectrum sunlight (see Maraccini et al., 2015 for solar simulator light spectra). Reactors were exposed to 6 h of full spectrum sunlight. Six hours of sunlight exposure was chosen based on previous data showing significant changes in gene expression at this exposure duration (McClary et al., 2017). The control reactor was kept in the dark at 15°C during the photoinactivation experiments. Both reactors were constantly stirred, and samples were taken from the reactors as described below. For experiments performed under anoxic conditions, an equal volume of nitrogen was injected into the reactors during sampling events to keep the reactors anoxic and at constant pressure. Triplicate experiments were performed in both oxic and anoxic conditions to generate three biological replicates for each condition.

### Culturability

To track *S. aureus* photoinactivation during experiments, 0.5-mL samples were taken from the experimental reactor every hour and from the control reactor every 3 h to determine culturability. Samples were diluted as necessary and appropriate dilutions were spread plated in duplicate on Brain Heart Infusion agar (BD Difco, Sparks, MD). After incubation at 37°C for 18–24 h, colonies were counted and sample concentrations were calculated in CFU/mL. Only dilutions resulting in countable colonies on duplicate plates were used to calculate sample concentrations. Inactivation rate constants were determined by non-linear regression using a biphasic first-order inactivation model:

$$\begin{array}{l} \ln\left(\frac{C}{C_{0}}\right)=\ln\left[\left(1-f\right)e^{-k_{1}F_{UVA+UVB}}+fe^{-k_{2}F_{UVA+UVB}}\right] \end{array}$$

where *ln(C/C*_0_*)* is the natural log-transformed relative concentration, *f* is the subpopulation fraction, *k*_1_ and *k*_2_ are the inactivation rate constants for the first and second phases, respectively, and *F*_*UVA*+*UVB*_ is fluence in kJ/m^2^. Fluence was calculated as has been done previously based on wavelengths in the UVA & UVB spectra (280–400 nm) (Maraccini et al., 2016a; McClary et al., 2017). Rate constants were also determined using log-linear and shoulder log-linear decay models (Geeraerd et al., 2005), but the biphasic model resulted in the best fit as determined by minimizing residual standard error and so was used for all subsequent analysis.

### RNA stabilization, extraction, and rRNA removal

At the end of each experiment (i.e., after 6 h of sunlight exposure), 200-mL samples were taken from both the experimental and control reactors for RNA extraction. Samples were immediately centrifuged for 10 min at 10,000 × *g*, and bacterial pellets were treated with RNAProtect Bacterial Reagent (Qiagen, Hilden, Germany). After 5 min of incubation at room temperature, samples were centrifuged again and the supernatant discarded. Stabilized bacterial pellets were stored at −80°C until RNA extraction.

RNA extractions were performed as described previously (McClary et al., 2017). In brief, stored bacterial pellets were resuspended in 0.2 mg/mL lysostaphin (Sigma-Aldrich, St. Louis, MO) and incubated at 37°C to lyse cells. Further lysis was performed by addition of a 100:1 vol:vol solution of Buffer RLT (Qiagen) and β-mercaptoethanol (Sigma-Aldrich), followed by bead beating in Lysis Matrix B tubes with a FastPrep-24 cell homogenizer (MP Biomedicals, Solon, OH). After brief centrifugation, lysate was transferred to new tubes, 470 μL ethanol was added to each sample, and RNA was extracted using the RNeasy Mini Kit (Qiagen), following the manufacturer's instructions. After elution in 60 μL of RNase-free water warmed to 60°C, extracts were DNase-digested using the RNase-free DNase Set (Qiagen), following the manufacturer's instructions. Samples were then cleaned up using the RNeasy Mini Kit, with final elution in 40 μL of RNase-free water warmed to 60°C. DNase digestion was confirmed by a qPCR assay targeting the *rexA* gene of *S. aureus* as described previously (McClary et al., 2017). Primer and probe sequences for the qPCR assay are provided in Table 1. For each set of extractions, an extraction blank was processed in parallel to verify lack of contamination from protocol reagents.

**Table 1:** Summary of primer and probe sequences used for RTqPCR reactions.

**Gene target**	**Gene name**	**Primer/probe**	**Sequence**	**Product size (bp)**
NWMN_0838	*rexA*	Forward primer	GATTTGGACTGACGCGCAA	142
		Reverse primer	ATCGACATCAATGCCATCACG	
		Probe	TGTTGCAGCCGCGGCAGGTTCAGGT	
NWMN_1240	*metL*	Forward primer	GCAGGCAGTTTAGCAACAGGTA	134
		Reverse primer	GAATCCATCATTTCCCGTGTTT	
		Probe	TGAATCAGATTTACACACATTGCCACCACA	
NWMN_1723	*hemY*	Forward primer	GAAGTCTGATAAAAGGTATGAAGGATGAG	122
		Reverse primer	TTCAATAAATGAGCTTAAACCATGCT	
		Probe	CCTGGCGCACCGAAAGGACAA	
NWMN_2341		Forward primer	CACCTGTTAAAGGTTCTGAATTTGC	122
		Reverse primer	CGCTTTAAACTTCTCATTGCTTACG	
		Probe	TCAACCTGCGCAACCATTTGAACG	
NWMN_2439	*cidB*	Forward primer	ACTGGCGTCATGCTGAATTTC	116
		Reverse primer	TCGATACCTACTGCGGCTGTT	
		Probe	ACGTCATTGTAACGTTATTGCCCCGATCT	

Total RNA samples were precipitated by adding 0.1 volume 3 M sodium acetate, 2.5 μL of 2 mg/mL glycogen, and 2.5 volumes 100% ethanol. The mixture was left overnight at −20°C before recovering precipitated RNA by centrifuging at 12,000 × *g* for 30 min at 4°C. RNA pellets were then washed twice in 1 mL ice cold 70% ethanol and recollected by centrifuging at 12,000 × *g* for 10 min at 4°C. After two ethanol washes, the RNA pellet was dissolved in 25 μL TE buffer. RNA precipitates were then depleted of rRNA using the MICROBExpress mRNA Enrichment Kit (Life Technologies, Carlsbad, CA), following the manufacturer's instructions. Five microliters from each extraction blank was also pooled and carried through the precipitation and rRNA-removal procedures as a negative control. Total RNA extracts, RNA precipitates, and rRNA-depleted samples were quantified on a Qubit v2.0 fluorometer or Nanodrop 1000, and RNA quality was confirmed on an Agilent 2100 Bioanalyzer at the Stanford Protein and Nucleic Acid Facility.

### Library preparation and sequencing

Indexed sequencing libraries were prepared from rRNA-depleted samples, including the negative control, using the ScriptSeq v2 RNA-Seq Library Preparation Kit and ScriptSeq Index PCR Primers (Epicentre, Madison, WI) following the manufacturer's instructions. PCR amplification of the indexed libraries was performed for 14 cycles. An additional positive control sample was also included, consisting of a 101 nt RNA sequence coding for a portion of the *grpE* gene of *Methanobacterium* sp. MB1, obtained from Integrated DNA Technologies (San Diego, CA). This sequence was chosen as a control as it would not be expected to occur in any of the experimental samples. Library preparation of the positive control followed the manufacturer's instructions for Severely Fragmented RNA. Amplified indexed libraries were quantified on an Agilent 2100 Bioanalyzer at the Stanford Functional Genomics Facility.

A total of 14 indexed libraries were generated, with each index corresponding to an individual sample (Table 2). The 12 oxic & anoxic sample libraries were combined in equimolar ratios to generate a pooled library. The positive control was added to the pooled library at a 10-fold lower molar ratio. The average volume of the oxic & anoxic sample libraries that were pooled was calculated, and this volume of negative control was also added to the pooled library. The pooled library was then sequenced on an Illumina MiSeq machine at the Stanford Functional Genomics Facility, generating 75 bp paired-end reads.

**Table 2:** Description of samples included for RNA sequencing.

**Sample number**	**Experiment number**	**Condition**	**Treatment**
1	1		Light
2			Dark
3	2	Oxic	Light
4			Dark
5	3		Light
6			Dark
7	4		Light
8			Dark
9	5	Anoxic	Light
10			Dark
11	6		Light
12			Dark
13	Positive control		
14	Negative control		

*Each sample corresponds to an individual treatment and condition, and each sample was indexed separately before pooling into a single sequencing library*.

### Sequencing data analysis

Raw sequencing data was demultiplexed and quality scored by Illumina MiSeq software to generate fastq files for forward and reverse reads of each indexed sample library. Initial read quality was assessed in FastQC version 0.11.4. Adapter trimming and quality filtering was performed for paired-end reads using Trimmomatic version 0.36 with provided adapter Fasta files for TruSeq3, removing low quality bases from the beginning and end of reads, and dropping reads shorter than 75% of the amplicon length or with quality scores <30 (Bolger et al., 2014). Following quality filtering, RNA-seq reads were aligned to the *S. aureus* genome using STAR version 2.5.3a with default settings (Dobin et al., 2013), and count matrices were generated from the alignment output using the Bioconductor GenomicAlignments package (Gentleman et al., 2004; Lawrence et al., 2013). The *S. aureus* genome and gene annotation information used for alignment and read counting, respectively, were obtained from Ensembl (taxid: 426430). Separate count matrices were generated for oxic and anoxic experiments, and each count matrix was filtered to remove genes with low or no counts (i.e., counts ≤1 across all samples) and to remove counts mapped to rRNA genes. Data from the count matrices were then analyzed using DESeq2 (Love et al., 2014). First, the regularized-logarithm (rlog) transformation was applied to the count matrices and used to calculate Euclidean distances between samples. Visualization of the sample-to-sample distances using a distance matrix revealed that samples from one experiment (Experiment #4, Samples 7 & 8, Table 2) were outliers (Supplementary Figure 1), and so this experiment was dropped from further analysis. Next, non-transformed count matrices were used to determine differential expression between light and dark conditions using DESeq2. DESeq2 is capable of evaluating differential expression on as few as two biological replicates (Love et al., 2014; Sekulovic and Fortier, 2015), making this method most appropriate for use in this study. Genes with a false discovery rate (FDR) <25% were considered significantly differentially expressed. After identifying differentially expressed genes, gene functions were explored using the KEGG pathways database. All sequencing data analysis was performed in Linux and R version 3.4.1. RNA-seq data are deposited in the NCBI sequence read archive (SRA) under accession number SRP125691.

Reverse transcription qPCR (RTqPCR) confirmation of RNA-seq results was performed for four selected genes: *metL, hemY, cidB*, and NWMN_2341 (Table 1). These genes were selected based on (1) their observed expression changes from RNA-seq data analysis, and (2) the ability to develop efficient qPCR assays for these genes. Differential expression between light and dark samples by RTqPCR was based on calculating a relative expression ratio (*R*) using the Pfaffl method (Pfaffl, 2001) with *rexA* as the reference gene. *rexA* was used as a reference because we previously developed an RTqPCR assay for this gene (McClary et al., 2017) and the RNA sequencing data analysis demonstrated that *rexA* was not significantly differentially expressed. Significant differential expression was determined if *R* was ≥ 2 or ≤ 0.5 and if *R* ± standard error (SE) did not include 1. Further details on RTqPCR assays are provided in the Supplementary Material.

## Results

### *Staphylococcus aureus* photoinactivation kinetics in oxic and anoxic conditions

**Figure 1:**
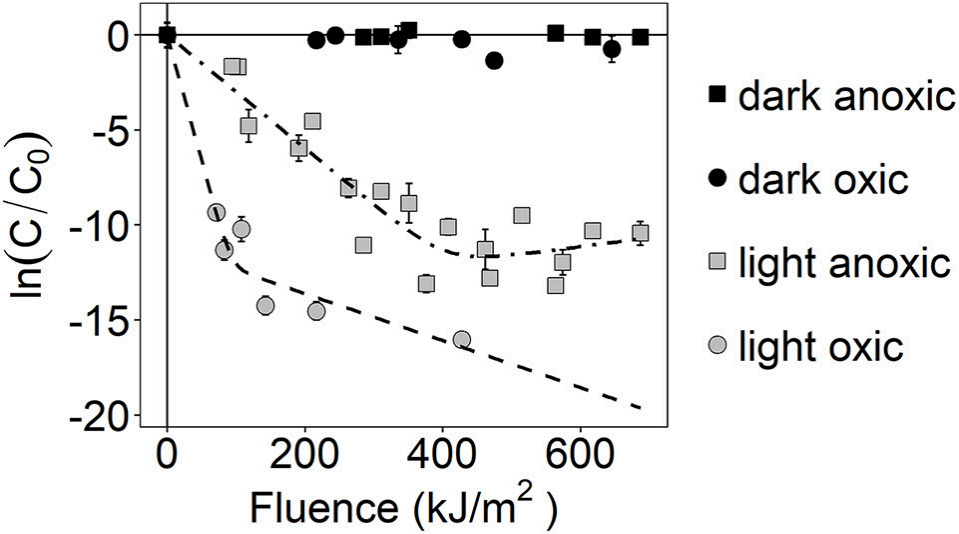
Photoinactivation kinetics of *S. aureus*, as measured by loss of culturability. ln(C/C_0_) is the natural-log transformed relative concentration. Error bars = ± standard deviation of technical replicates. Dashed lines are modeled biphasic inactivation curves.

Inactivation of *S. aureus* was observed during sunlight exposure under both oxic and anoxic conditions, as shown in Figure 1, and was discussed in our previous publication (McClary et al., 2017). Inactivation kinetics are biphasic under both conditions, displaying relatively fast inactivation followed by a period of slow or no inactivation. Non-linear regression was used to fit the observed data to biphasic first-order inactivation curves, and inactivation rate constants are presented in Table 3. The first-order rate constant during the initial phase of inactivation was larger in the oxic compared to anoxic condition (*k*_1_ ± SE = 0.1 ± 0.01 m^2^ kJ^−1^ in oxic conditions vs. 0.03 ± 0.002 m^2^ kJ^−1^ in anoxic; Z-test, *P* < 0.05). These rate constants are in agreement with those presented in our previous work (McClary et al., 2017). The first-order rate constants during the second phase of inactivation (*k*_2_) were 0.01 ± 0.005 m^2^ kJ^−1^ and −0.005 ±0.007 m^2^ kJ^−1^ in oxic and anoxic conditions, respectively. *S. aureus* continued to slowly decay following the initial rapid decay in oxic conditions. In anoxic conditions, *k*_2_ is not different from 0. No inactivation was observed in dark controls, suggesting that all observed inactivation was due to sunlight exposure. After 6 h of sunlight exposure [i.e., fluence (*F*_*UVA*+*UVB*_) between 427 and 687 kJ/m^2^], the concentration of cultivatable cells was at or below the limit of detection (i.e., ≤ 20 CFU/mL) in oxic experiments and was ~700 CFU/mL for anoxic sunlight experiments. After 6 h of dark incubation, the concentration of cultivatable cells in the control oxic and anoxic experiments remained steady at ~10^7^ CFU/mL. These samples were used to investigate gene expression changes in sunlight-exposed experiments vs. dark controls.

**Table 3:** Modeled inactivation rate constants of *S. aureus* under sunlight exposure.

**Condition**	**Inactivation rate constants (m**^**2**^ **kJ**^**−1**^**)**	
	***k_1_***	***k_2_***
Oxic	0.1 ± 0.01	0.01 ± 0.005
Anoxic	0.03 ± 0.002	−0.005 ± 0.007

*Inactivation was fit to a biphasic model, and reported rate constants represent the first (k_1_) and second (k_2_) phases of inactivation*.

### Differential gene expression due to sunlight exposure in oxic and anoxic conditions

RNA sequencing was used to investigate changes in *S. aureus* gene expression as a result of sunlight exposure under oxic and anoxic conditions. A summary of sample-specific data generated by RNA sequencing is presented in Table 4. Sequencing resulted in ~21 million total reads, with an average of ~900,000 read pairs per sample. Quality filtering removed between 7 and 39% of read pairs per sample, and the resulting filtered reads aligned to the *S. aureus* genome at rates of at least 92%. As described in the Materials and Methods, based on Euclidean sample-to-sample distances generated from rlog-transformed count matrices, samples from one experiment (Experiment #4) clustered far from all other samples; samples from this experiment were subsequently removed from further gene expression analyses.

**Table 4:** Summary of sample-specific data generated by RNA sequencing.

**Condition**	**Oxic**						**Anoxic**					
**Experiment number**	**1**		**2**		**3**		**4**		**5**		**6**	
**Sample**	**Light**	**Dark**	**Light**	**Dark**	**Light**	**Dark**	**Light**	**Dark**	**Light**	**Dark**	**Light**	**Dark**
Data generated (MB)	480	405	468	340	316	392	171	423	467	306	157	368
Total read pairs	1157836	971815	1132694	823982	843365	950993	412592	1022242	1127263	748010	430231	891451
Total read pairs after Trimmomatic	1077294	906926	1035527	752140	567485	867647	385046	943652	1044373	665765	260350	816798
Reads mapped (%)	98.7	99.1	99.2	99	97.3	98.3	99.1	97.4	99.4	98.8	92.2	94.5

To determine the effects of sunlight exposure on gene expression, differential expression analysis was carried out comparing sunlight-exposed samples from a single experimental condition (oxic or anoxic) to corresponding controls prepared identically and kept in the dark. Using this framework, a total of 71 differentially expressed genes were identified from oxic experiments (Table 5) and 18 from anoxic experiments (Table 6). Of these, three genes were differentially expressed under sunlight exposure in both the oxic and anoxic conditions: NWMN_1608 was increased in expression, while *ebpS* and NWMN_0867 were decreased in expression. Under both conditions, most differentially expressed genes showed reduced expression under sunlight-exposed conditions compared to the dark control; nine genes and two genes were significantly increased in abundance in sunlit oxic and anoxic conditions, respectively. Of the total number of differentially expressed genes, the proportions of genes showing increased expression under oxic and anoxic conditions are similar.

**Table 5:** List of significantly differentially expressed genes from oxic experiments.

**Gene name**	**Gene description**	**Fold change**	**FDR (%)**
*hemY*	Protoporphyrinogen oxidase	5.97	3.1
NWMN_1978	Conserved hypothetical protein	5.64	13.0
NWMN_0650	Conserved hypothetical protein	4.50	5.5
NWMN_1466	Conserved hypothetical protein	4.16	7.9
*vraB*	Acetyl-CoA C-acetyltransferase VraB	3.57	9.3
NWMN_1608	Conserved hypothetical protein	3.54	5.0
NWMN_2520	Conserved hypothetical protein	3.35	5.3
*narG*	Nitrate reductase, alpha subunit	2.78	18.8
*glk*	Glucokinase	2.70	13.0
*thrS*	Threonyl-tRNA synthetase	0.46	21.6
*gudB*	NAD-specific glutamate dehydrogenase	0.43	20.2
NWMN_1689	Conserved hypothetical protein	0.42	14.5
*agrC*	Staphylococcal accessory gene regulator protein C	0.41	12.1
NWMN_1806	Conserved hypothetical protein	0.39	24.8
NWMN_2026	Aldehyde dehydrogenase family protein	0.38	12.6
*glnA*	Glutamine synthetase	0.38	8.6
*gapR*	Glycolytic operon regulator	0.36	13.0
*citC*	Isocitrate dehydrogenase, NADP-dependent	0.36	19.2
NWMN_1263	Aconitate hydratase	0.35	21.7
NWMN_0845	ATP-dependent Clp protease, ATP-binding subunit ClpB	0.34	5.7
NWMN_1529	ATPase AAA family protein	0.34	6.4
NWMN_2210	Formate dehydrogenase homolog	0.31	4.4
*sdhA*	Succinate dehydrogenase flavoprotein subunit	0.31	6.5
NWMN_0377	Conserved hypothetical protein	0.31	5.3
*glmS*	Glucosamine-fructose-6-phosphate aminotransferase, isomerizing	0.30	23.8
*rnbP*	RNase P RNA component class B	0.30	9.0
NWMN_0475	Cysteine synthase homolog	0.30	18.4
NWMN_0250	ABC transporter, permease protein	0.28	2.8
*spoVG*	Stage V sporulation protein G homolog	0.28	8.8
NWMN_0460	Conserved hypothetical protein	0.28	14.2
NWMN_2262	Conserved hypothetical protein	0.27	7.9
*gapA*	Glyceraldehyde 3-phosphate dehydrogenase 1	0.27	2.4
*pdhD*	Dihydrolipoamide dehydrogenase: subunit E3	0.26	2.4
*pgm*	2,3-bisphosphoglycerate-independent phosphoglycerate mutase	0.26	5.3
*spa*	Immunoglobulin G binding protein A precursor (protein A)	0.26	8.0
NWMN_1195	Conserved hypothetical protein	0.26	6.1
*dnaK*	Chaperone protein DnaK	0.26	3.2
*sarA*	Staphylococcal accessory regulator A	0.25	13.0
NWMN_0585	Conserved hypothetical protein	0.25	8.8
*pdhC*	Dihydrolipoamide acetyltransferase component of pyruvate dehydrogenase complex	0.25	2.5
NWMN_0163	Conserved hypothetical protein	0.24	9.9
NWMN_1371	Conserved hypothetical protein	0.24	7.2
NWMN_0366	Conserved hypothetical protein	0.24	6.4
NWMN_2392	Conserved hypothetical protein	0.24	12.6
NWMN_2282	Conserved hypothetical protein	0.23	5.0
NWMN_1477	Conserved hypothetical protein	0.23	10.1
*clfA*	Clumping factor A	0.22	1.8
*hutG*	Formiminoglutamase	0.22	1.8
NWMN_0735	Conserved hypothetical protein	0.22	5.0
NWMN_2088	Conserved hypothetical protein	0.22	9.9
*ebpS*	Elastin binding protein	0.22	0.5
NWMN_2597	Conserved hypothetical protein	0.22	8.8
*citZ*	Citrate synthase II	0.21	9.9
NWMN_2548	Conserved hypothetical protein	0.21	3.2
*qoxC*	Quinol oxidase polypeptide III	0.21	2.4
NWMN_2087	Conserved hypothetical protein	0.21	11.1
*katA*	Catalase	0.20	2.0
*poxB*	Pyruvate oxidase	0.20	2.4
*tpi*	Triosephosphate isomerase	0.19	0.5
NWMN_2086	Alkaline shock protein 23	0.19	2.5
NWMN_1746	Conserved hypothetical protein	0.19	2.5
*cidB*	Holin-like protein CidB	0.18	0.5
NWMN_1631	Conserved hypothetical protein	0.18	2.5
NWMN_0783	CsbD-like superfamily protein	0.17	1.1
NWMN_1526	Hypothetical protein	0.17	1.5
NWMN_0867	Conserved hypothetical protein	0.16	0.7
NWMN_1989	Conserved hypothetical protein	0.12	3.5
NWMN_0721	Sigma 54 modulation protein	0.11	2.5
NWMN_1527	Conserved hypothetical protein	0.11	0.5
NWMN_2406	Conserved hypothetical protein	0.11	0.9
NWMN_0868	Conserved hypothetical protein	0.07	0.5

**Table 6:** List of significantly differentially expressed genes from anoxic experiments.

**Gene name**	**Gene description**	**Fold change**	**FDR (%)**
NWMN_2341	NAD dependent epimerase/dehydratase family protein	8.30	7.4
NWMN_1608	Conserved hypothetical protein	2.17	19.6
NWMN_1804	Conserved hypothetical protein	0.38	19.6
*mnhA*	Na+/H+ antiporter, MnhA component	0.30	19.6
*atpA*	ATP synthase F1, alpha subunit	0.30	7.4
NWMN_0470	Polyribonucleotide nucleotidyltransferase	0.27	19.6
NWMN_1123	Conserved hypothetical protein	0.27	7.4
NWMN_1800	Conserved hypothetical protein	0.26	11.3
NWMN_1008	Conserved hypothetical protein	0.23	7.5
NWMN_0759	Conserved hypothetical protein	0.21	0.3
NWMN_0867	Conserved hypothetical protein	0.21	12.6
*ebpS*	Elastin binding protein	0.19	9.1
*metL*	Homoserine dehydrogenase	0.19	3.4
NWMN_0860	Conserved hypothetical protein	0.16	0.4
NWMN_0748	Conserved hypothetical protein	0.14	12.6
NWMN_1913	Conserved hypothetical protein	0.13	9.1
NWMN_1004	Conserved hypothetical protein	0.13	0.1
NWMN_1101	Conserved hypothetical protein	0.09	0.1

### Functional classification of differentially expressed genes

**Figure 2:**
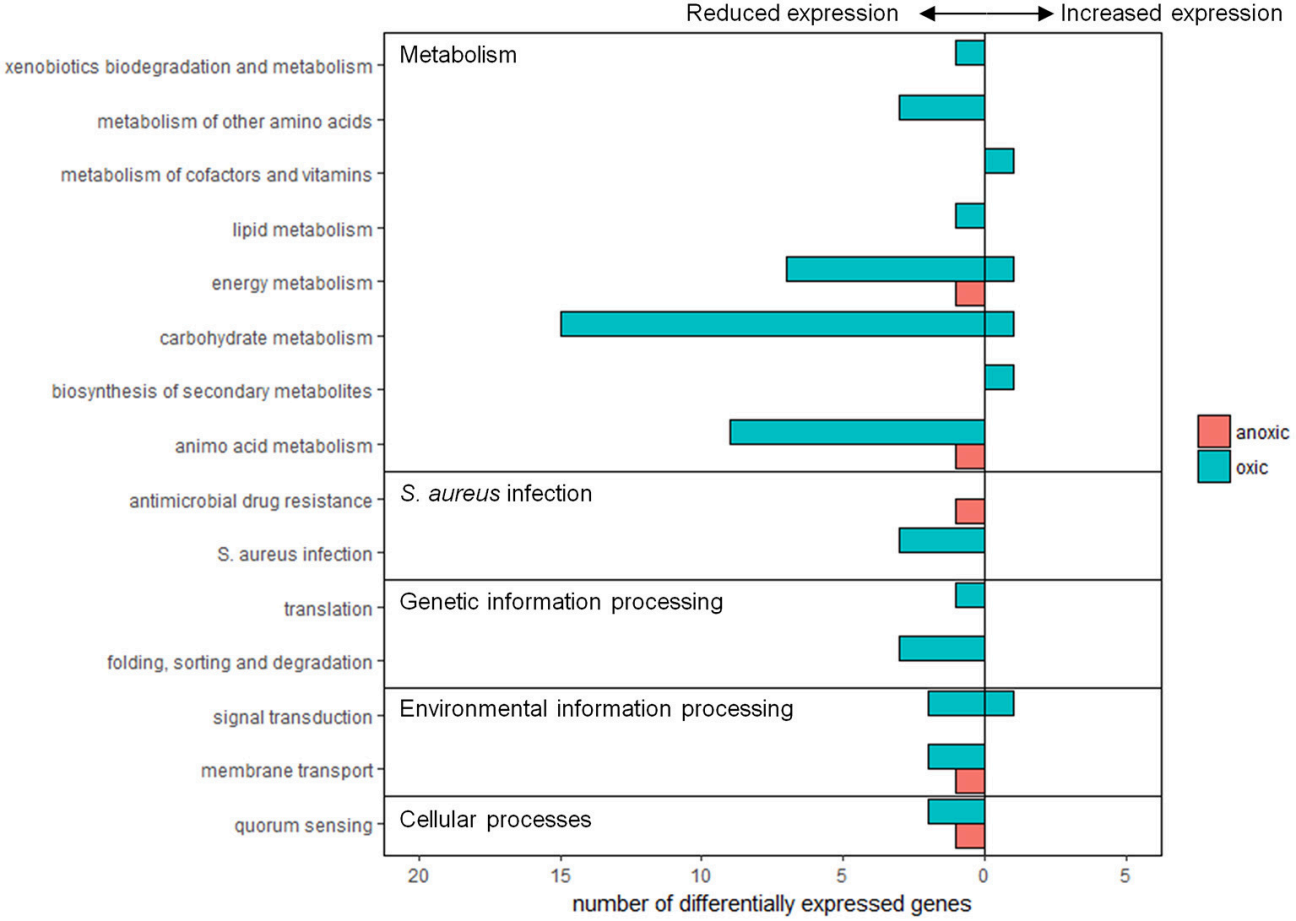
Summary of differentially expressed genes assigned to functional groups according to KEGG pathways. Pink bars represent expression from anoxic experiments, and blue bars represent expression from oxic experiments. Values to the left and right of the y-axis indicate genes with reduced or increased expression, respectively.

The genome of *S. aureus* subsp. *aureus* str. Newman contains genes encoding 2,624 proteins, of which 1,051 are classified as hypothetical meaning that their function is unknown or unconfirmed. In the oxic condition, 30 differentially expressed genes (42% of 71) were assigned to functional pathways whereas for the anoxic condition, three differentially expressed genes (17% of 18) were assigned (Figure 2). Functional pathways with decreased expression due to sunlight exposure in both the oxic and anoxic conditions involved metabolism, environmental information processing, genetic information processing, cellular processes, and human disease. Expression of other genes involved in metabolism and environmental information processing were also induced by sunlight exposure in the oxic condition. Neither of the genes induced by sunlight exposure in the anoxic condition was assigned to functional pathways in KEGG.

### Differential gene expression not categorized to functional pathways

Differentially expressed genes not assigned to pathways include genes with no annotated function or with predicted functions not yet linked to specific *S. aureus* cell reactions or networks. In the oxic condition, 39 genes were differentially expressed but not assigned to KEGG functional pathways. Five hypothetical proteins showed increased expression; the remaining 34 differentially expressed genes not assigned to functional pathways in the oxic condition were decreased in expression following sunlight exposure. These included a glycolytic operon regulator (*gapR*), a subunit of Clp protease (NWMN_0845), an ATPase family protein (NWMN_1529), a component of RNase P (*rnbP*), an ABC transporter (NWMN_0250), a sporulation protein (*spoVG*), staphylococcal accessory regulator A (*sarA*), elastin binding protein (*ebpS*), an alkaline shock protein (NWMN_2086), holin-like protein CidB (*cidB*), a CsbD-like superfamily protein (NWMN_0783), sigma 54 modulation protein (NWMN_0721), and 22 hypothetical proteins.

In the anoxic condition, 15 genes were differentially expressed and not assigned to functional pathways. These included an epimerase/dehydratase family protein (NWMN_2341), a Na+/H+ antiporter (*mnhA*), a polyribonucleotide nuleotidyltransferase (NWMN_0470), and 12 hypothetical proteins. Of these, NWMN_2341 and a conserved hypothetical protein (NWMN_1608) were increased in expression; the expression of the remaining 13 genes was decreased following sunlight exposure.

### Confirmation of gene expression with RTqPCR

**Figure 3:**
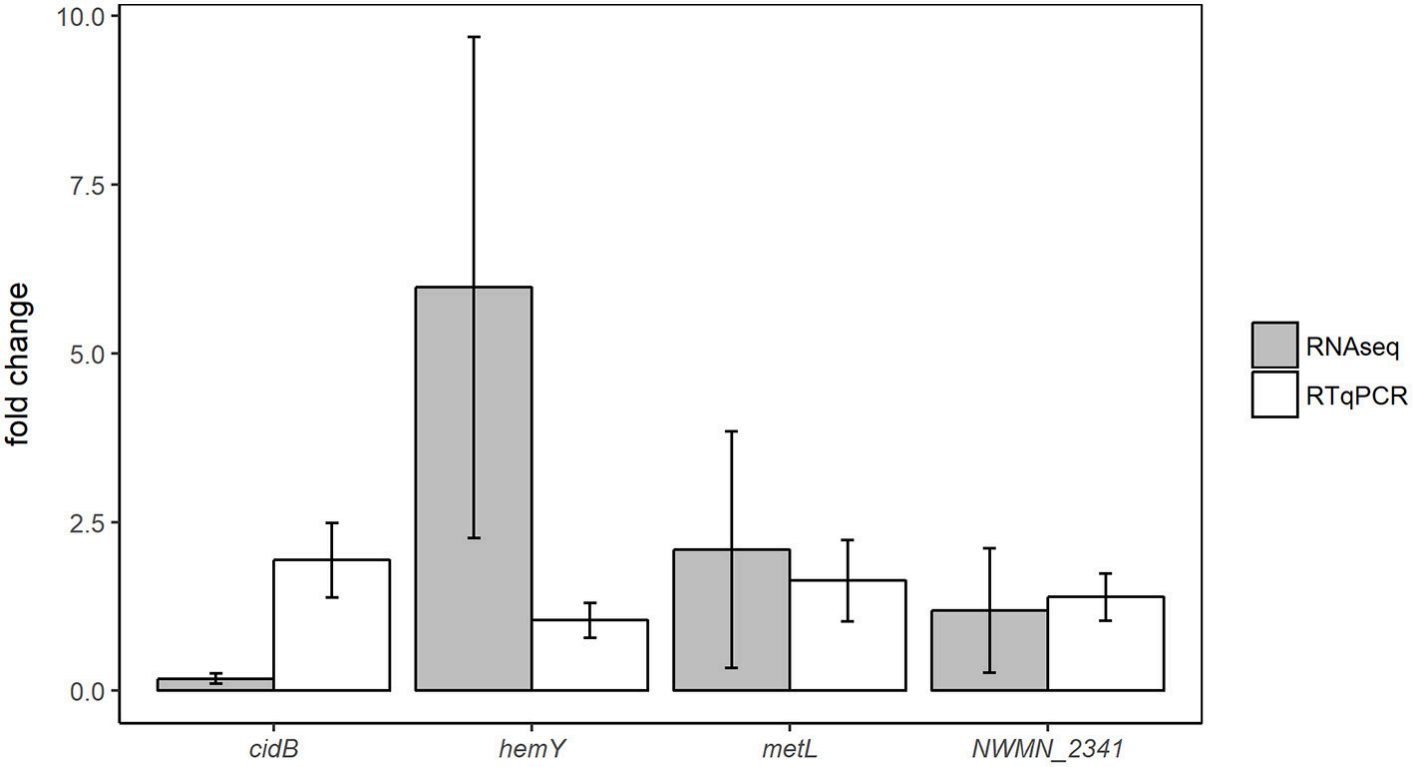
Comparison of differential expression results from oxic experiments using RNA-seq or RTqPCR. Error bars = ± SE.

**Figure 4:**
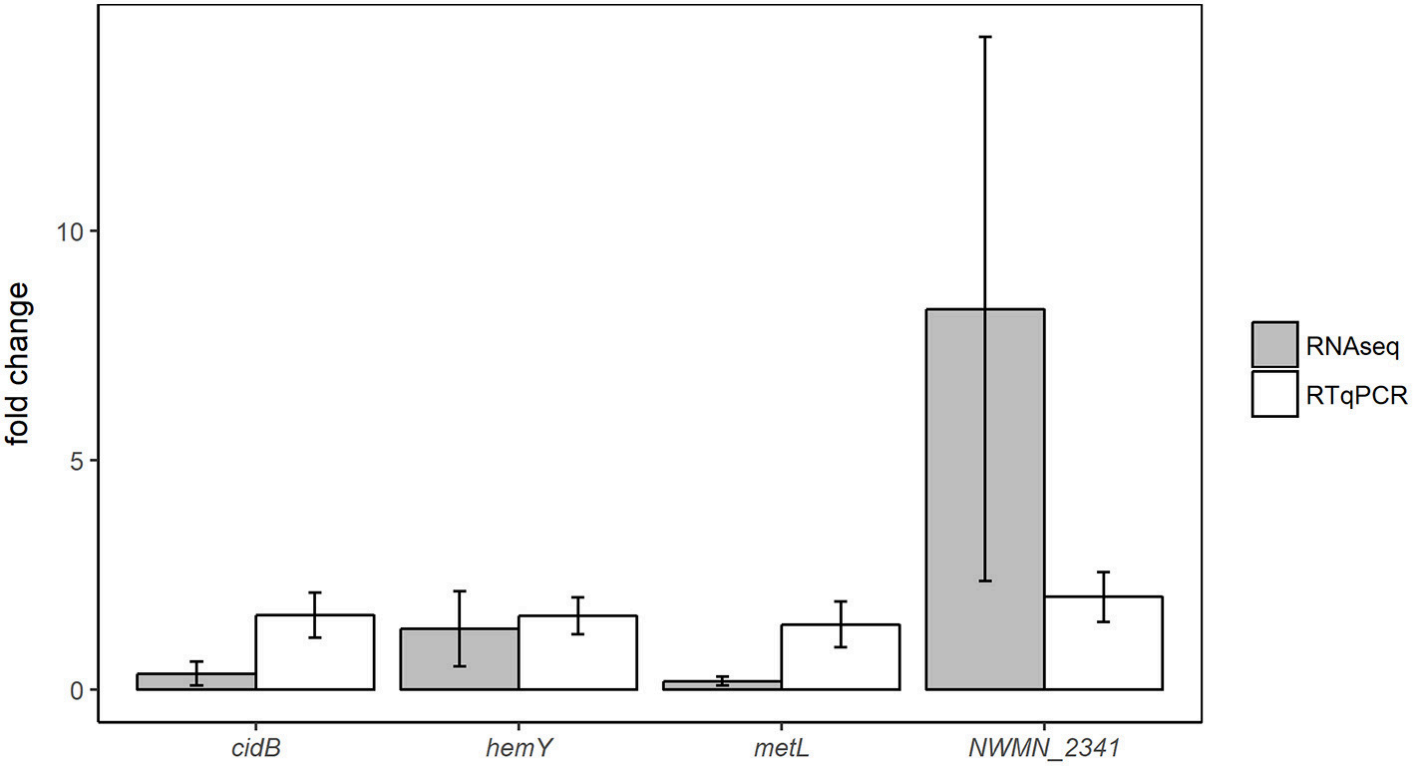
Comparison of differential expression results from anoxic experiments using RNA-seq or RTqPCR. Error bars = ± SE.

Expression changes in the same samples analyzed by RNA sequencing were also measured using RTqPCR assays targeting four different genes: *cidB, hemY, metL*, and NWMN_2341. Fold changes of these genes detected by RTqPCR and RNA sequencing are shown for the oxic and anoxic cases in Figures 3, 4, respectively. As RTqPCR and RNA-seq use different methods to normalize the “baseline” expression level in samples, we opted not to compare the specific fold change values but rather to compare whether statistical analysis of each method concluded an increase, decrease, or no change in expression of the gene of interest. With this treatment of the data, RTqPCR and RNA-seq results were in agreement in most cases: 2/4 genes are in agreement in the oxic condition and 3/4 genes are in agreement in the anoxic condition. Exceptions were for *metL* in the anoxic samples, and *cidB* and *hemY* in the oxic samples. RNA sequencing detected significant decreases in expression for *metL* in the anoxic condition and *cidB* in the oxic condition, whereas RTqPCR did not detect any significant expression changes. Similarly, RNA sequencing detected a significant increase in expression of *hemY* in the oxic condition, while the fold change generated by RTqPCR was not significant. Others have also found that RTqPCR results do not always agree with RNA-seq or microarray results, usually in cases where significance is detected by one method but not by the other (Song et al., 2016; Al-Jassim et al., 2017).

## Discussion

To better understand the ways in which *S. aureus* responds to oxygen-mediated and non-oxygen-mediated photoinactivation, we used RNA sequencing to identify gene expression changes between oxic and anoxic sunlit reactors and their corresponding dark controls. After 6 h of sunlight exposure, concentrations of cultivatable *S. aureus* were reduced by more than four orders of magnitude in both oxic and anoxic conditions, and were reduced to levels at or below the limit of detection in the sunlit oxic treatment. Despite significant reduction in cultivatable cell concentration after 6 h of sunlight exposure, our previous work showed only slight reduction in the intact cell concentration during the same exposure period, as measured by fluorescence microscopy (McClary et al., 2017). The combination of intact cell membranes and detectable mRNA concentrations in these samples suggests the possibility that *S. aureus* entered a viable but non-culturable (VBNC) state under the sunlight stress condition, and these metrics have been used in previous studies to conclude the presence of VBNC cells (Liu et al., 2009; Chaisowwong et al., 2012; Pasquaroli et al., 2013). Additionally, samples collected after 6 h, which were analyzed by RNA sequencing, were collected during the second phase of the observed biphasic inactivation. This second phase of inactivation is often assumed to represent a resistant subpopulation of the bacterial community, a shift to a resistant phenotype, and/or a shift to a VBNC state, which could be triggered by environmental stresses (Brouwer et al., 2016). While the existence of a VBNC state is generally accepted within the scientific community, there remains uncertainty regarding what specific metrics must be used to define this state and differentiate from other non-growing states (Hammes et al., 2011; Ramamurthy et al., 2014; Pinto et al., 2015). Future work to characterize the transition of *S. aureus* into a VBNC state during sunlight exposure should include attempts at resuscitation of non-culturable cells.

To identify gene expression changes associated with oxic and anoxic photostress conditions, we used RNA sequencing and differential expression analysis with DESeq2 to compare mRNA transcript abundances between sunlight-exposed samples and control samples under either oxic or anoxic conditions, separately. To identify significant differential expression, we chose to consider genes identified by the DESeq2 program with FDR < 25%. Significant expression thresholds based on FDR are highly variable among previous microarray and RNA-seq studies, often ranging between 5 and 30%, while other studies base results on nominal *p*-values without correction for multiple hypothesis testing (Graham et al., 2005; Bore et al., 2007; Stasiewicz et al., 2011; Dhanjal et al., 2014; Sassoubre et al., 2014). We opted to consider significance based on FDR due to the importance of multiple hypothesis testing in detecting gene expression changes across the full genome, and we chose to set a somewhat liberal threshold at FDR < 25% based on our goals in this study to identify and explore overall transcriptional response to photostress conditions.

Overall, we identified 71 and 18 genes which were significantly differentially expressed after 6 h of sunlight exposure in oxic and anoxic conditions, respectively. This is comparable to the number of differentially expressed genes identified in *E. faecalis* during sunlight exposure using microarrays (Sassoubre et al., 2014), but is a smaller amount of genes than those identified in *E. coli* during sunlight exposure using RNA sequencing (Al-Jassim et al., 2017). Of the genes identified as differentially expressed, most showed significantly decreased expression in sunlight exposed reactors compared to their dark controls: 87 and 89% in oxic and anoxic conditions, respectively. Due to the fact that experiments were performed in oligotrophic conditions, it is possible that *S. aureus* in the sunlit experiments were forced to shut down transcription of cell functions not immediately necessary for combating the damaging effects of sunlight. In contrast, while control dark reactors were similarly oligotrophic, *S. aureus* in these reactors were exposed only to starvation stress and therefore were able to maintain a higher level of transcription in contrast to the sunlight-exposed cells. Additionally, sunlight exposure may lead to the direct mutation and degradation of mRNA transcripts in the sunlight-exposed samples. While the effects of UVA+UVB exposure on DNA have been more comprehensively investigated (Sinha and Häder, 2002; Rastogi et al., 2010), UVA+UVB can lead to degradation of RNA molecules through similar mechanisms (Swenson and Setlow, 1964; Qiao and Wigginton, 2016). It is therefore possible that mRNA transcripts were able to persist longer in the dark control reactors than in the sunlight-exposed reactors, and this differential persistence could also have an effect on the overall decreased gene expression detected in sunlight-exposed reactors. Another factor that may have influenced the overall changes in gene expression is a transition to a viable but non-culturable state. As mentioned previously, samples collected following sunlight exposure exhibited substantially reduced culturable cell numbers compared to those in dark controls. However, our previous work demonstrated that *S. aureus* cells remain intact in these samples (McClary et al., 2017), suggesting that cells remain viable but may be transitioning to a non-culturable state in the sunlight-exposed system. The difference between non-culturable cells in the sunlight-exposed samples and largely culturable cells in the dark control samples could control some of the transcriptome changes observed.

Due to the significant losses in *S. aureus* culturability observed after 6 h of sunlight exposure, genes identified with increased expression in the sunlight-exposed reactors relative to dark controls are hypothesized to be of great importance to the *S. aureus* photostress response. For the oxic case, genes with increased expression included *hemY, vraB, narG, glk*, and five conserved hypothetical proteins. The gene *hemY*, which was expressed in the oxic sunlight-exposed experiments ~6-fold more than in the dark controls, codes for a protoporphyrinogen oxidase and is involved in porphyrin metabolism. Porphyrins are well-known photosensitizers, and the use of synthetic or naturally occurring porphyrins for the enhancement of photoinactivation in applications like photodynamic therapy has been studied for many years (Jori and Brown, 2004; Ferro et al., 2007; Khlebtsov et al., 2013; Nakonieczna et al., 2016). Specifically, hemY catalyzes the oxidation of protoporphyrinogen (or coproporphyrinogen), yielding protoporphyrin (or coproporphyrin) and hydrogen peroxide. Despite the fact that this reaction yields potentially damaging hydrogen peroxide as well as the photosensitizer protoporphyrin, the enhancement of protoporphyrinogen oxidase activity would be required to metabolize and subsequently reduce the overall levels of endogenous porphyrins. A previous study in mice found that the use of a protoporphyrinogen oxidase inhibitor led to the buildup of endogenous porphyrin molecules and subsequently enhanced the effects of photodynamic therapy (Fingar et al., 1997). Additionally, in *Bacillus subtilis*, a Gram-positive bacterium with very similar hemY structure to that of *S. aureus* (Lobo et al., 2015), hemY mutants were found to accumulate endogenous coproporphyrin (Hansson and Hederstedt, 1994). In contrast, a recent study found that activation of hemY led to increased photosensitization in *S. aureus* (Surdel et al., 2017). Interestingly, of the four *S. aureus* strains tested in that study, activation of hemY in *S. aureus* Newman led to the least significant reduction in cell viability following light exposure (Surdel et al., 2017). We therefore hypothesize that oxygen-mediated indirect photoinactivation mechanisms in *S. aureus* are strongly dependent on levels of endogenous photosensitizers within the cells, and that the metabolism of photosensitizing porphyrins is potentially a more efficient stress response method under starvation conditions than the expression of antioxidant enzymes. This hypothesis should be explored in future work using mutants for specific genes in the porphyrin metabolism pathway, such as *hemY*, or by quantifying and identifying intracellular porphyrins (Nitzan and Kauffman, 1999; Fyrestam et al., 2015).

In addition to the increased expression of *hemY, S. aureus* also increased expression of *vraB, narG*, and *glk* following exposure to sunlight in oxic conditions. *vraB* codes for an acetyl-CoA acetyltransferase and is involved in the TCA cycle. Expression of *vraB* in *S. aureus* was previously found to be induced by other stresses, including treatment with the antibacterial compound berberine chloride (Wang et al., 2008) and exposure to Cr(VI) (Zhang et al., 2014), suggesting expression of *vraB* could be important for general *S. aureus* stress response. *narG* codes for the alpha subunit of nitrate reductase, a membrane-bound oxidoreductase enzyme. While *narG* is typically only regulated during anaerobic metabolism (Richardson et al., 2001), nitrate can also serve as an important precursor to reactive oxygen species like hydroxyl radical (Brezonik and Fulkerson-Brekken, 1998). *S. aureus* may therefore increase expression of *narG* in order to manage the potentially damaging effects of nitrate to the cell. *S. aureus* also increased expression of *glk*, coding for glucokinase, following sunlight exposure in oxic conditions. Glucokinase is involved in a range of metabolic functions, including metabolism of galactose and sucrose, as well as the biosynthesis of streptomycin. While overall more metabolism genes were observed to be decreased in expression following sunlight exposure, the increased expression of *glk* suggests that *S. aureus* remains metabolically active. Future work to identify *S. aureus* metabolism of specific substrates following sunlight exposure is warranted.

In the sunlit anoxic treatments, fewer genes were identified as differentially expressed. This could be because bacteria in anoxic experiments had been exposed to less overall stress due to the fact that oxygen-mediated photostress was not present in these systems. *S. aureus* in the anoxic experiments also decayed more slowly and better tracked the cell numbers in the dark controls, further pointing to the anoxic treatment being less stressful than the oxic. However, despite the fact that fewer differentially expressed genes were identified, we would like to stress the fact that, by using true biological replicates and carefully considered metrics of significant expression, the genes identified as differentially expressed are likely those that show the greatest expression changes and are most consistently differentially expressed in the anoxic photostress condition.

In the anoxic condition, two genes were identified as significantly increased in expression: NWMN_2341, coding for a NAD dependent epimerase/dehydratase family protein, and NWMN_1608, coding for a conserved hypothetical protein identified as a probable membrane transporter according to the UniProt database. NWMN_1608 is also the only gene identified as significantly increased in expression during sunlight exposure in both the oxic and anoxic conditions, suggesting its importance for the *S. aureus* photostress response. The increased expression of a probable membrane transporter could indicate that *S. aureus* are responding to membrane damage, or that the cells are attempting to increase the removal of toxic species from inside the cell. Cell membrane damage due to sunlight exposure could occur in an anoxic environment due to non-ROS radicals generated from endogenous cell components or direct UV damage of intermembrane proteins (Oppezzo et al., 2001; Kalisvaart, 2004). Our previous work suggests that sunlight exposure in anoxic conditions does lead to increased membrane damage in *S. aureus* (McClary et al., 2017). Additionally, previous work on the photostress response of *E. coli* confirmed the importance of efflux pumps in protecting *E. coli* from critical damage (Al-Jassim et al., 2017).

In conclusion, we have investigated gene expression changes associated with oxic and anoxic photostress in *S. aureus* in clear oligotrophic seawater. Results suggest that the photostress responses associated with oxygen-mediated and non-oxygen-mediated photoinactivation mechanisms are different from each other. Additionally, the increased expression of *hemY* in the oxic photostress condition suggests the importance of porphyrin metabolism for combating oxygen-mediated photoinactivation. While further work is needed to confirm that the gene expression changes described here correspond to protein level changes as well, this study helps to identify genes of importance for responding to different types of photostress. In particular, future work should focus on improving our understanding of types and concentrations of endogenous photosensitizers present in bacterial pathogens and fecal indicators, as these appear to play an important role in photoinactivation.

## Author contributions

AB and JM conceived and designed the study; JM wrote the manuscript, conducted experiments, and analyzed the data; AB and JM edited the manuscript; AB supervised the project; AB and JM read and approved the final manuscript.

### Conflict of interest statement

The authors declare that the research was conducted in the absence of any commercial or financial relationships that could be construed as a potential conflict of interest.
