# Supplementary material for: Transcriptional Response of Staphylococcus aureus to Sunlight in Oxic and Anoxic Conditions
Source: Front Microbiol. 2018 Feb 23;9:249. doi: 10.3389/fmicb.2018.00249 (PMC5863498; doi:10.3389/fmicb.2018.00249)
Supplement: Supplementary file 1 [file DataSheet1.PDF]

Supplementary Material

Transcriptional response of *Staphylococcus aureus* to sunlight in oxic and anoxic conditions

Jill S. McClary, Alexandria B. Boehm*

*** Correspondence:** Alexandria B. Boehm: aboehm@stanford.edu

1 Supplementary materials and methods

To confirm RNA-seq results, RTqPCR was used to quantify transcript levels of four selected genes: *metL*, *hemY*, *cidB*, and NWMN_2341. *rexA* was used as an internal reference, and the RTqPCR assay for *rexA* has been previously described (McClary et al., 2017). All primers and probes were developed using Primer Express v3.0 (Applied Biosystems, Foster City, CA) and NCBI Primer-BLAST and were supplied by Integrated DNA Technologies (San Diego, CA). Primer and probe sequences are provided in Table 1 of the main text. All assay cycling parameters and efficiencies were optimized using *Staphylococcus aureus* DNA extracts. RTqPCR reactions were performed on an Applied Biosystems StepOnePlus instrument using the TaqMan RNA-to-C_T *1-Step* kit (Life Technologies, Carlsbad, CA). All reactions were performed in 20-µL volumes and consisted of 1X TaqMan RT-PCR Mix, 1X TaqMan RT Enzyme Mix, and 2 µL RNA template. For *metL* and *cidB*, reactions consisted of 0.8 µM forward primer, 0.8 µM reverse primer, and 0.15 µM probe. For *hemY*, reactions consisted of 0.5 µM forward primer, 0.5 µM reverse primer, and 0.25 µM probe. For NWMN_2341, reactions consisted of 0.2 µM forward primer, 0.2 µM reverse primer, and 0.15 µM probe. Cycling parameters for all reactions were as follows: 15 minutes at 50 °C, 10 minutes at 95 °C, and 40 cycles of 15 seconds at 95 °C followed by 1 minute at 60 °C. Results were generated in Applied Biosystems StepOne software v2.3 with a threshold set at 0.03.

2 Supplementary Figures

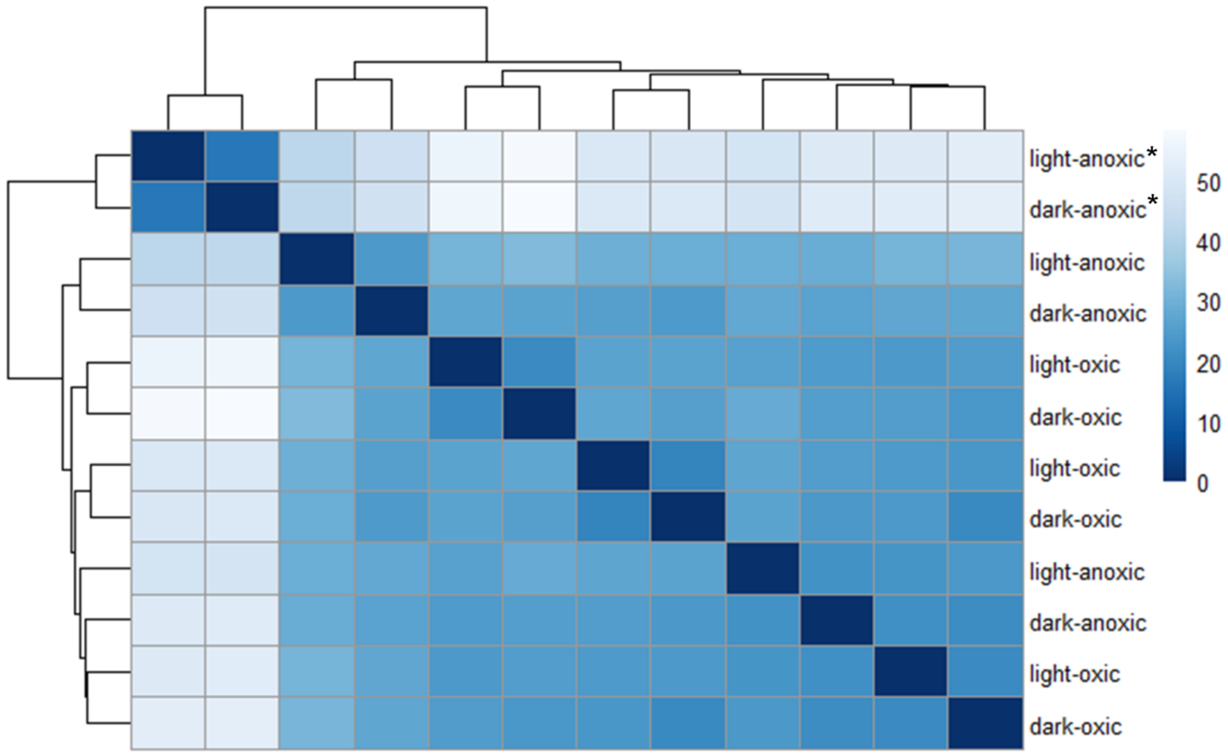

Supplementary Figure 1. Heatmap representing the sample-to-sample Euclidean distances from rlog-transformed count matrices. Samples marked by * were removed from RNA-seq differential expression analysis.
